# Taxonomic study of the genus *Prorophora* Ragonot, 1887 (Lepidoptera, Pyralidae, Phycitinae) in China, with description of a new species

**DOI:** 10.3897/zookeys.180.2615

**Published:** 2012-04-05

**Authors:** Jiayu Liu, Houhun Li

**Affiliations:** 1 College of Life Sciences, Nankai University, Tianjin 300071, P. R. China

**Keywords:** Lepidoptera, Pyralidae, Phycitinae, *Prorophora*, new species, new record, China

## Abstract

The genus *Prorophora* Ragonot, 1887 is newly recorded for China. Of the three species treated here, *Prorophora (Reisserempista) binacantha* **sp. n.** is described as new; *Prorophora (Prorophora) albidogilvella* Roesler, 1970 and *Prorophora (Reisserempista) mongolica* Roesler, 1970 are diagnosed and newly recorded for China. Images of adults and illustrations of genital structures are provided, along with a key to the known species.

## Introduction

*Prorophora* was established by Ragonot in 1887, with *Prorophora curvibasella* Ragonot, 1887 as the type species from Namangan, Turkestan (now Uzbekistan). Following Ragonot, Hampson (1912) described *Prorophora dialeuca* from Sri Lanka and Marion (1957) described *Prorophora grisealella* from Senegal. Roesler (1970) establishedthe subgenus *Reisserempista*, with *Prorophora (Reisserempista) mongolica* Roesler, 1970 as the type species. The same author (1973) revised part of the Phycitinae species of the Palaearctic Region, in which he treated *Aproceratia* Amsel, 1950 as a synonym of *Epischidia* Ragonot, 1901, and transferred the latter genus to *Prorophora* as a subgenus.Roesler (1973) proposed a system of three subgenera: *Prorophora* Ragonot, 1887, *Epischidia* Ragonot, 1901 and *Reisserempista* Roesler, 1970, based on the characters of the maxillary palp, the male antenna and the female antrum. As *Epischidia* Ragonot, 1901 is both a homonym and a synonym of *Epischidia* Rebel, 1901 (Fletcher and Nye 1984), Roesler (1987) substituted *Prorophora (Aproceratia)* Amsel, 1950 for *Prorophora (Epischidia)* Ragonot. Later on, Falkovitsch (1999) described *Prorophora halothamni* from Uzbekistan and Asselbergs (2004) described *Prorophora (Prorophora) kazachstaniella* from Kazakhstan. To date, the genus *Prorophora* comprisesthree subgenera with twelve valid species, which occur in North Africa, Southeast Europe and Asia.

In the present paper, we report three species from China based on the specimens collected in Inner Mongolia Autonomous Region, Gansu Province and Ningxia Hui Autonomous Region. A key to all the known species, diagnoses for *Prorophora* Ragonot, 1887 and subgenera *Reisserempista* Roesler, 1970 and *Prorophora* Ragonot, 1887 are provided. The new species *Prorophora binacantha* sp. n. is described in the subgenus *Reisserempista*. The type specimens are deposited in the Insect Collection, College of Life Sciences, Nankai University, Tianjin, China.

## Taxonomic accounts

### Prorophora

Ragonot, 1887

http://species-id.net/wiki/Prorophora

Prorophora Ragonot, 1887: 252. Type species: *Prorophora curvibasella* Ragonot, 1887, by monotypy.
Aproceratia Amsel, 1950: 224. Type species: *Proceratia rhectogramma* Meyrick, 1937 (= *Myelois albunculella* Staudinger, 1879), by monotypy. Synonymised by Roesler (1973).
Reisserempista Roesler, 1970: 55. subgenus of *Prorophora* Type species: *Prorophora (Reisserempista) mongolica* Roesler, 1970, by monotypy.

#### Diagnosis.

*Prorophora* is characterized by the frons with a distinct laterally compressed projection (Fig. 2). It is similar to *Gymnancyla* Zeller, 1848, but can be distinguished by the following characters: the maxillary palp absent or discernible; the transtilla tiny thorn-shaped if present, the 8th sternum is rarely extended in the male genitalia; and the antrum usually sclerotized strongly in the female genitalia. In *Gymnancyla* Zeller, the maxillary palp is developed, reaching end of the second segment of the labial palp; the transtilla is usually triangular, the 8th sternum is distinctly extended in the male genitalia; and the antrum is inconspicuous or weakly sclerotized in the female genitalia.

**Distribution.** Mongolia, Russia (Ural), Uzbekistan, Kazakhstan, Turkey, Iraq, Iran, Afghanistan, Pakistan, Lebanon, Palestine, Egypt, Sudan, Senegal and Sri Lanka. Newly recorded for China (Gansu, Inner Mongolia, Ningxia).

#### Key to species of the genus

**Table:** 

1	Maxillary palp discernible	2
–	Maxillary palp absent (subgenus *Prorophora*)	8
2	Male antennal segments 3−9 curved; female antrum with scent scale tuft posterolaterally(subgenus *Reisserempista*)	3
–	Male antennal segments 3−9 not curved; female antrum without scent scale tuft posterolaterally (subgenus *Aproceratia*)	4
3	Valva with one sclerotized band extending from below base of costa to about 2/3 of ventral margin; signum extending from entrance to posterior 1/3 of corpus bursae (Figs 6, 9)	*Prorophora (Reisserempista) binacantha* sp. n.
–	Valva without sclerotized band; signum being a small sclerotized subrounded plate (Roesler, 1973: fig. 25) (Figs 7, 10)	*Prorophora (Reisserempista) mongolica*
4	Costa 2/3 length of valva; corpus bursae with one or two sclerotized plates at entrance	5
–	Costa about as long as valva; corpus bursae without plate at entrance	7
5	Gnathos with lateral arms widening from base to apex	6
–	Gnathos with lateral arms widening in posterior half (Falkovitsch, 1999: fig. 8)	*Prorophora (Aproceratia) halothamni*
6	Phallus with one cornutus, transtilla absent; corpus bursae with two sclerotized plates at entrance (Roesler, 1973: fig. 21)	*Prorophora (Aproceratia) albunculella*
–	Phallus with five cornuti, transtilla membranous; corpus bursae with one sclerotized plate at entrance (Roesler, 1973: fig. 22)	*Prorophora (Aproceratia) eberti*
7	Costa furcate at apex, valva without sclerotized band; antrum longer than wide, apophyses anteriores longer than apophyses posteriores (Roesler, 1973: fig. 23)	*Prorophora (Aproceratia) afghanella*
–	Costa acuate at apex, valva with one sclerotized band extending from below base of costa to near end of ventral margin; antrum as long as wide, apophyses anteriores shorter than apophyses posteriores (Roesler, 1973: fig. 24)	*Prorophora (Aproceratia) senganella*
8	Forewing with an obvious longitudinal white stripe	9
–	Forewing without obvious longitudinal white stripe	10
9	Forewing with a white stripe extending from middle of upper margin of cell to termen (Hampson, 1912: pl. G, fig. 34)	*Prorophora (Prorophora) dialeuca*
–	Forewing with a wide white stripe extending from base to postmedian line along costal margin (Marion, 1957: pl. 1, fig. 5)	*Prorophora (Prorophora) grisealella*
10	Phallus without, or with one cornutus	11
–	Phallus with more than two cornuti	12
11	Culcita one pair, sacculus straight at apex; signum hemispheroidal, with a sclerotized plate at entrance of corpus bursae (Roesler, 1973: fig. 19) (Figs 5, 8, 11)	*Prorophora (Prorophora) albidogilvella*
–	Culcita absent, sacculus acuate at apex; signum ovate, without sclerotized plate at entrance of corpus bursae (Roesler, 1973: fig. 18)	*Prorophora (Prorophora) curvibasella*
12	Phallus with two cornuti (Roesler, 1973: fig. 20)	*Prorophora (Prorophora) sacculicornella* Roesler
–	Phallus with three to five cornuti (Asselbergs, 2004: fig. 7)	*Prorophora (Prorophora) kazachstaniella*

### Subgenus Reisserempista

Roesler, 1970

#### Diagnostic characters.

Maxillary palp present. Male antenna with basal 3−9 flagellomeres curved, flagellomeres 5−9 each with one thorn on dorsal surface; culcita absent. Female antrum with a pair of scent scale tufts posterolaterally(shed easily), accessory sac present.

### Prorophora (Reisserempista) binacantha sp. n.

urn:lsid:zoobank.org:act:1DC86CF1-34DC-444A-B50F-AB36D78A6101

http://species-id.net/wiki/Prorophora_binacantha

Figs 1 –3 6 9

#### Type material.

Holotype ♂ – **China, Inner Mongolia Autonomous Region:** Mt. Helan (38.8°N, 105.7°E), Alxa Zuoqi, 1683 m, 29.VII.2010, coll. Hongxia Liu and Zhiwei Zhang. Paratypes: 1 ♂, 1 ♀, Erenhot (43.6°N, 112.0°E), 960 m, 02.VIII.2002, coll. Zhiqiang Li and Dandan Zhang, genitalia slide nos. LJY10019 ♂, LJY10289 ♀; 1 ♂, 1 ♀, Buyant (41.8°N, 107.0°E), Urad Houqi, 1075 m, 17.VIII.2006, coll. Zhiwei Zhang, genitalia slide nos. LJY10292 ♂, LJY11034 ♀; 30 ♂♂, 15 ♀♀, Mt. Helan (38.8°N, 105.7°E), Alxa Zuoqi, 1683−1836 m, 29.VII.−03.VIII.2010, coll. Hongxia Liu and Zhiwei Zhang, genitalia slide nos. LJY10658 ♂, LJY11031 ♂, LJY11028 ♀; **Ningxia Hui Autonomous Region:** 1 ♂, Yinchuan (38.4°N, 106.2°E), VI.1986, genitalia slide no. LJY10028 ♂; 5 ♂♂, Suyukou (38.7°N, 105.9°E), Mt. Helan, 2000 m, 10.VIII.2005−09.VIII.2006, coll. Xinpu Wang, Feng Yang and Qi He, genitalia slide nos. LJY09037 ♂, LJY09065 ♂, LJY10195, ♂.

#### Diagnosis.

This species is similar to *Prorophora (Reisserempista) mongolica* Roesler, 1970, but can be distinguished by the following characters: the forewing dark brown along veins between antemedian and postmedian lines; in the male genitalia, the valva with one spine at ventral 2/3, and the phallus with 3−5 cornuti; in the female genitalia, the elongate signum extending from the entrance to posterior 1/3 of the corpus bursae. In *Prorophora (Reisserempista) mongolica*, the forewing is yellowish brown along veins between antemedian and postmedian lines; the valva lacks the ventral spine, and the phallus has two cornuti; the signum is a small sclerotized subrounded plate, located in posterior 1/4 of the corpus bursae.

#### Description.

**Figure 1.**
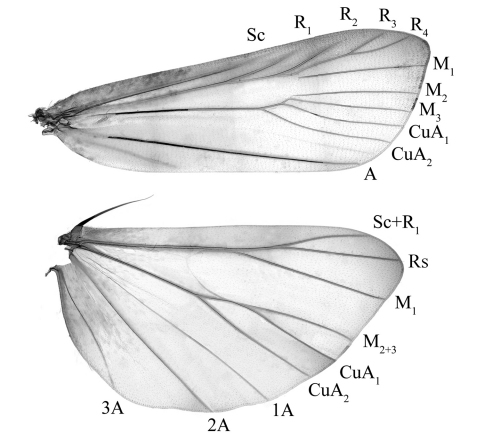
Wing venation. *Prorophora (Reisserempista) binacantha* sp. n., paratype, slide No. LJY10292W.

**Figures 2–5.**
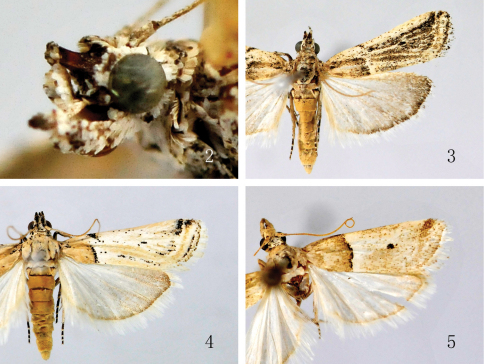
Adults of *Prorophora* spp. **2** Headof *Prorophora (Reisserempista) binacantha* sp. n. (lateral view), paratype, male **3** *Prorophora (Reisserempista) binacantha* sp. n., holotype, male **4** *Prorophora (Reisserempista) mongolica*, male**5** *Prorophora (Prorophora) albidogilvella*, female.

Adult (Figs 1, 2, 3) with wingspan 16.0−19.0 mm. Vertex greyish white, with two longitudinal short black stripes. Antennal scape greyish brown to dark brown, 2.5−3.0 times as long as wide; flagellum dorsally greyish white ringed with yellowish brown, ventrally overall yellowish brown; dense cilia on ventral surface as long as wide of flagellum. Labial palp with first and second segments greyish white, mixed with brown and dark brown; third segment dark brown, about 1/3 length of second. Proboscis yellowish brown, greyish white at base. Patagium, thorax and tegula greyish white mixed with dark brown. Forewing: venation (Fig. 1); ground coloration pale greyish brown, dark brown along veins between antemedian and postmedian lines; antemedian line greyish white, extending from costal 1/3 to dorsal 2/5, obliquely straight, edged with a broad dark brown band along inner side posteriorly, with a thin dark brown band along outer side anteriorly; discocellular stigmata brownish black, clearly separated; postmedian line greyish white, curved slightly inward at middle, edged with a broad dark brown band along inner side, with a thin yellowish brown band along outer side; termen pale dark brown; cilia greyish white. Hindwing greyish brown, outer margin dark brown; cilia greyish white. Legs with femura and tibiae greyish white, mixed with black; tarsi dark brown, mixed greyish white, ringed with greyish white at apex of each tarsomere. Abdomen pale yellow to yellowish brown dorsally, grayish white ventrally, mixed with dark brown.

**Figures 6–8.**
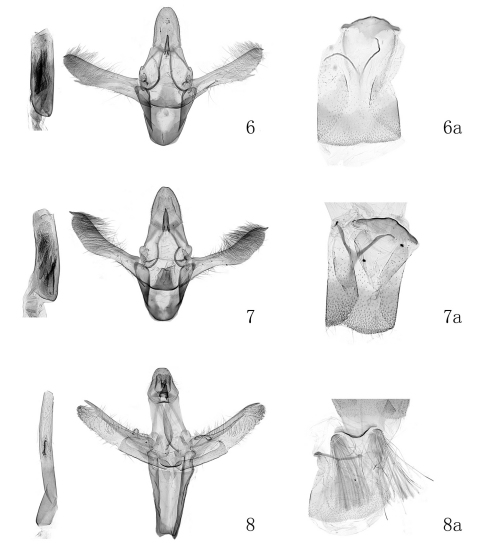
Male genitalia of *Prorophora* spp. **6** *Prorophora (Reisserempista) binacantha* sp. n., paratype, slide No. LJY09037 **7** *Prorophora (Reisserempista) mongolica*, slide No. LJY10022 **8** *Prorophora (Prorophora) albidogilvella*, slide No. LJY09075 **6a–8a** 8th abdominal segment and culcita.

**Male genitalia** (Fig. 6). Uncus broad tongue-shaped, wide at base, narrowed slightly toward blunt apex, length 1.2−1.5 times basal width. Gnathos about half length of uncus. Transtilla present as a pair of sclerotized tiny plates. Valva with dorsal and ventral margins nearly parallel, utmost width about 1/5 length; sclerotized band extending from below base of costa to about ventral 2/3, then produced to a strong free apical spine. Costa slightly exceeding end of valva, produced to a small down-curved apical spine. Clasper as long as gnathos, covered with sparse fine setae, ear-shaped, dentate along outer margin; sacculus slender, about 2/5 length of valva. Vinculum longer than 3/5 length of valva, rounded anteriorly. Juxta trapezium-shaped; anterolateral side extending outward, gradually sharpened, curved backward. Phallus stout, obviously shorter than valva, almost full of sclerotized thorns; cornuti composed of 3−5 sclerotized unequally lengthened thorns, placed medially, longest one about 1/4 length of phallus.

**Figures 9–11.**
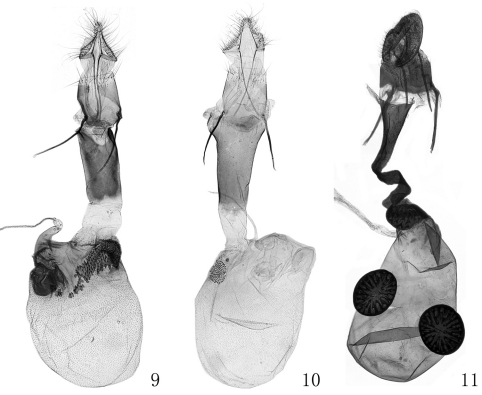
Female genitalia of *Prorophora* spp. **9** *Prorophora (Reisserempista) binacantha* sp. n., paratype, slide No. LJY10289 **10** *Prorophora (Reisserempista) mongolica*, slide No. LJY10018 **11** *Prorophora (Prorophora) albidogilvella*, slide No. LJY11081.

**Female genitalia** (Fig. 9). Papillae anales subtriangular, round posteriorly. Apophyses anteriores slightly shorter than apophyses posteriores. Antrum nearly as wide as eighth tergum, about twice as long as wide, parallel sided, concave medially on posterior margin. Ductus bursae straight, as wide as antrum, about 2/3 length of antrum. Corpus bursae ovate, membranous, about twice as long as antrum; signum being a sclerotized elongate plate with dense thorns, extending from entrance to posterior 1/3 of corpus bursae, narrowing gradually; accessory sac arising from posterior 1/3 of corpus bursae, with a few scattered thorns basally; ductus seminalis from posterior end of accessory sac.

#### Distribution.

 China (Inner Mongolia, Ningxia).

#### Etymology.

 The specific name is derived from the Latin prefix *bin*-(= two, double), and *acanthus* (= spinous), referring to the valva having an apical spine on the costa and a strong free apical spine on the ventral margin.

### Prorophora (Reisserempista) mongolica

Roesler, 1970

http://species-id.net/wiki/Prorophora_mongolica

Figs 4 7 10

Prorophora (Reisserempista) mongolica Roesler, 1970: 55; Roesler, 1973: 76; Roesler, 1987: 394. [Holotype: ♂, Chovd aimak, Mongolia, deposited in Hungarian National Museum, Budapest, Hungary].

#### Material examined.

**China, Inner Mongolia Autonomous Region:** 1 ♂, Chengguanzhen, Dengkou County (40.3°N, 107.0°E), 1000 m, 19.VIII.2002, coll. Zhiqiang Li and Dandan Zhang; 31 ♂♂, 15 ♀♀, Buyant (41.8°N, 107.0°E), Urad Houqi, 1075 m, 17.VIII.2006, coll. Zhiwei Zhang; 2 ♂♂, 4 ♀♀, Mt. Helan (38.8°N, 105.7°E), Alxa Zuoqi, 1836 m, 03.VIII.2010, coll. Hongxia Liu and Zhiwei Zhang; **Gansu Province:** 1 ♀, Minqin County (38.6°N, 103.0°E), 1343 m, 26.VII.2006, coll. Xinpu Wang and Xiangfeng Shi. (genitalia slide nos. LJY09048 ♂; LJY10022 ♂; LJY10027 ♂; LJY11032 ♂; LJY09036 ♀; LJY10018 ♀; LJY11030 ♀).

#### Diagnosis.

Adult (Fig. 4) with wingspan 15.0−18.0 mm.This species is characterized by the forewing with yellowish brown basal field edged with black on outer margin posteriorly, pale yellowish brown along veins between antemedian and postmedian lines; the costa exceeding end of valva and produced to an apical spine curved backward, the phallus with two cornuti in the male genitalia (Fig. 7); the corpus bursae densely covered with tiny spines, the subrounded signum located at posterior 1/4 of the corpus bursae, and the ductus seminalis from posterior margin of the corpus bursae near the ductus bursae in the female genitalia (Fig. 10).

#### Distribution.

 China (Inner Mongolia, Gansu); Mongolia.

### Subenus Prorophora

Ragonot, 1887

#### Diagnostic characters.

 Maxillary palp absent. Male culcita absent or one pair. Female antrum elongate; two signa prominent on surface of corpus bursae, covered with conical spines on inner surface; ductus seminalis from posterior part of corpus bursae.

### Prorophora (Prorophora) albidogilvella

Roesler, 1970

http://species-id.net/wiki/Prorophora_albidogilvella

Figs 5 8 11

Prorophora albidogilvella Roesler, 1970: 50. [Holotype: ♂, Gobi Altaj aimak, Mongolia, deposited in Hungarian National Museum, Budapest, Hungary].
Prorophora (Prorophora) albidogilvella Roesler, 1970: Roesler, 1973: 65; Roesler, 1987: 394.

#### Material examined.

**China, Inner Mongolia Autonomous Region:** 1 ♂, 4 ♀♀, Ejin Qi (41.9°N, 101°E), 927 m, 17−18.VII.2006, coll. Xinpu Wang and Xiangfeng Shi; **Gansu Province:** 1 ♂, Minqin County (38.6°N, 103.0°E), 1343 m, 26.VII.2006, coll. Xinpu Wang and Xiangfeng Shi. (genitalia slide nos. LJY09075 ♂; LJY11074 ♀; LJY11081 ♀).

#### Diagnosis.

Adult (Fig. 5) with wingspan 15.0−18.0 mm. This species is conspicuously different from its congeners by the costa distally thornlike and separated from the valva, and the apex-straight sacculus with a dorsoapical spine in the male genitalia (Fig. 8); and by the ductus bursae curved in S shape distally, and having a sclerotized ring-shaped plate at the entrance of the corpus bursae which is covered with pyramidlike thorns on inner surface in the female genitalia (Fig. 11).

#### Distribution.

 China (Inner Mongolia Autonomous Region, Gansu); Mongolia.

## Supplementary Material

XML Treatment for Prorophora

XML Treatment for Reisserempista

XML Treatment for Prorophora (Reisserempista) binacantha

XML Treatment for Prorophora (Reisserempista) mongolica

XML Treatment for Prorophora

XML Treatment for Prorophora (Prorophora) albidogilvella
